# The KT Jeang Retrovirology prize 2020: Tatyana Golovkina

**DOI:** 10.1186/s12977-020-00529-x

**Published:** 2020-07-20

**Authors:** Retrovirology Editorial

**Affiliations:** London, UK

**Fig. 1:**
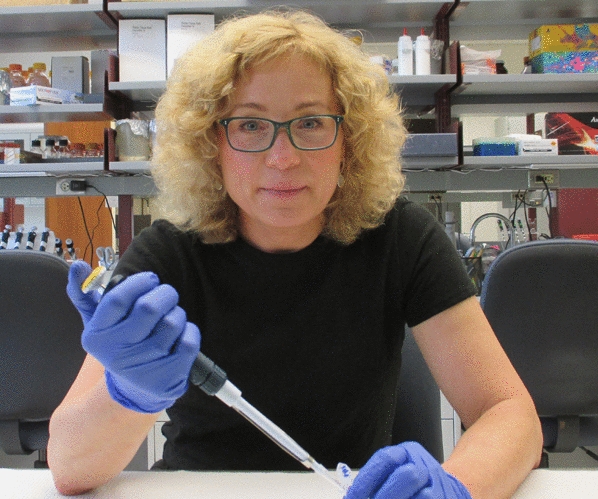
Tatyana Golovkina at the bench

A native of Russia, Tatyana Golovkina graduated in 1985 with a MS in biology from Moscow State University (Fig. 1). While it marked the beginning of an exciting and fulfilling journey into the world of scientific research, it was not the career path she and her family envisioned. Like many young people in the former USSR, Golovkina spent much of her youth training to become a pianist. This required countless hours of practice which became increasingly difficult with the arrival of a baby sister in her family’s small apartment. To her family’s dismay, she decided it would be better to pursue a different career and relegate piano to a hobby; the problem was that none of them had envisioned anything else. So, perhaps as her first scientific experiment, she began a process of elimination to rule out careers she didn’t want to do, which finally singled out biology.

This deliberation paid off, however, as Golovkina resolved to attend what was the best university in the Soviet Union at the time. In Russia, biology majors traditionally start by learning a broad range of disciplines, from botany to vertebrate and invertebrate biology, and then funnel down to a more specialized discipline. Golovkina found that she most enjoyed learning about microorganisms, specifically viruses. Virology holds a special place of pride in Russia, since Dmitri Ivanovsky discovered the first virus, tobacco mosaic virus, which was causing a disease ravaging tobacco plants in Crimea, in 1892.

Golovkina completed her undergraduate and master’s studies at Moscow State University and moved to the Cancer Research Center in Moscow to pursue her Ph.D. Working at the Institute of Carcinogenesis, she continued her studies of virology, as many viruses of course were being studied for their roles in carcinogenesis. She joined the lab of Irina Kryukova, a retrovirologist known for her work on the chicken retrovirus known as Rous Sarcoma Virus. Golovkina credits Kryukova with teaching her that in order to be successful as a scientist, you have to be willing to be exploratory, as long as you are asking interesting questions and follow the science wherever it may lead. Golovkina also worked in the lab of Andrey Gudkov as she continued her graduate studies on the evolution of endogenous retroviruses in the genomes of small mammals, specifically rodents.

In 1990, Golovkina graduated with a Ph.D. in oncology at a momentous time in the history of the Soviet Union. As political reforms swept the country and its international borders were opened to citizens for the first time, many Russian scientists used the opportunity to leave the country and pursue their careers elsewhere. Gudkov helped find jobs for all the members of his lab who wanted to leave, and Golovkina landed a postdoctoral position in the lab of Susan Ross in the Department of Biochemistry at the University of Illinois at Chicago (UIC).

At UIC in Ross’ lab, Golovkina studied genetic elements of the mouse mammary tumor virus (MMTV) that direct expression of the genes in tissue specific fashions, specifically in the mammary gland. However, she soon realized that she was most interested in studying host–pathogen interactions. Ross encouraged her to start series of experiments with MMTV, which led to Golovkina’s first significant publication [1]. In that study, Ross, Golovkina and their co-authors, including her future husband, Alexander Chervonsky, showed that transgenic mice expressing viral superantigen (Sag) derived from of an exogenous MMTV had no Sag-specific T cells and were protected from infection with the same virus. This indicated that MMTV utilizes cells of the immune system in its infection pathway, and mice that retain endogenous MMTVs resist exogenous viruses expressing Sags of the same T cell specificity.

In 1994, Golovkina moved with Ross to the Cancer Center and Department of Microbiology at the University of Pennsylvania. It was there that Golovkina realized that the traditional discipline of virology alone was not enough to answer the many questions she had begun to ponder related to host–pathogen interactions. She started learning more about immunology and genetics to understand how the immune system responds to pathogens, and how the genetic composition of a given host makes it resistant or susceptible to particular viruses. These questions led her to take a new position as an independent investigator at The Jackson Laboratory, where she took advantage of their vast mouse genetic resource and worked for the next 8 years studying susceptibility and resistance to viral infections in various strains of mice. During this time she published another foundational paper [2], in which she and her colleagues showed that when mice of the I/LnJ strain were infected with certain mouse retroviruses, they produced a highly potent neutralizing response that interferes with viral infection and blocked the ability of the virus to escape.

In 2005, Golovkina moved to the University of Chicago along with her husband, joining the tenured faculty in the Department of Microbiology, where her lab has continued to focus on host–pathogen interactions. Specifically, they are studying how the innate immune system detects retroviral infection and initiates virus-neutralizing adaptive immune responses, and the mechanisms evolved by retroviruses to overcome host protective responses. To investigate these important questions, her group employs virus-resistant mice capable of controlling retroviruses from distinct genera. Using mice from retrovirus-resistant strains and mouse retroviruses, in 2011, Golovkina’s team found that endosomal Toll-like receptor 7 (TLR7) is an innate immune receptor that detects mouse retroviruses and signals to stimulate the production of virus-neutralizing antibodies [3].

Most viruses enter the host through surfaces exposed to commensal bacteria that protect the host from incoming pathogens. In 2011, Golovkina’s team performed pioneering work showing that a mucosally transmitted mouse retrovirus, mouse mammary tumor virus (MMTV), exploits gut commensal bacteria for sufficient transmission and spread. Specifically, they demonstrated that the MMTV virion-associated Gram-negative bacterial wall component lipopolysaccharide (LPS) activates the Toll-like receptor 4 (TLR4), stimulating production of the immunosuppressive cytokine IL-10, aiding virus evasion of the host immune response [4]. In 2015, they showed how during its budding, this enveloped retrovirus acquires the host’s LPS binding receptors to attach LPS to its virions [5].

In a completely independent study conducted by Julie Pfeiffer’s group at University of Texas Southwestern, it was shown that human picornavirus and reovirus transmission also depends on the gut microbiota [6]. Since these two independent discoveries, more evidence of the microbiome’s role in viral infections continued to mount. Specifically, noroviruses and rotaviruses, among others, have since been added to the list of viruses known to interact with gut microbiota (for review, see [7]).

Golovkina continued her work studying how some hosts are resistant to persistent viral infections while others remain susceptible. A long journey into forward genetics using positional cloning and other methods culminated in 2017 when her group discovered that the specific allele of MHC Class II gene H2-Ob in I/Ln J mice supports the production of virus-neutralizing antibodies [8]. H2-Ob (known as DOB in humans) together with H-2a (DOA in humans) forms a constitutive heterodimer, known as H2-O (DO in human) and is a negative regulator of the MHC Class II immune response, but I/LnJ mice have a mutation that limits its function. I/LnJ mice mount a vigorous response to retroviral infections that helps keep chronic retroviruses in check. Certain alleles of human DOA and DOB were subsequently linked to the control or persistence of human infections with hepatitis B and C viruses ([8] and Graves et al., J Immunology, in press).

That 2017 study was the result of nearly 20 years of work, a matter of patience and persistence Golovkina credits with keeping her lab running through the early days as a young researcher when she struggled to secure funding. Experiments with animal models can be time-consuming and expensive, often taking years of painstaking research to yield results. From the teachings of Kryukova, Ross, and other mentors, along with Chervonsky’s constant partnership, Golovkina learned that she needed to maintain multiple projects in parallel, any one of which might lead to interesting scientific conclusions. She begins most of her research projects at the bench herself to determine whether or not a particular project is worth pursuing. If so, she can then hand it off to her students and postdocs, and then repeat the cycle with another project. This continuous supplement of new ideas is crucial when it may take up to 20 years to find the right answer. In addition, Golovkina really likes to be at the bench, which she still truly enjoys.

Golovkina thinks that the definitions of scientific disciplines like microbiology, immunology, genetics, or neurobiology are becoming obsolete as new tools and technologies allow scientists to cross these traditional boundaries. If there is an interesting scientific question, researchers now have the ability to delve into other fields and connect with experts to help them answer it.

During her career, Golovkina has advised over 20 undergraduate and graduate students, two postdoctoral scholars, and has taught many courses in virology and microbiology. She credits her teaching style to one of her former teachers, Vadim Agol, an eminent Russian virologist who lectured from simple written note cards and a blackboard. She thinks that in an age when students are constantly immersed in their laptops, smartphones, and digital media, this simple strategy helps engage students in a more direct way.

Golovkina has served on numerous committees at UChicago and is a member of the American Society for Microbiology. In 2018, she was named as a Fellow of the American Academy of Microbiology, and in 2019 as a Fellow of the American Association for the Advancement of Science.
